# Implant Strength Contributes to the Osseointegration Strength of Porous Metallic Materials

**DOI:** 10.1115/1.4065405

**Published:** 2024-05-13

**Authors:** Elizabeth Mathey, Matthew H. Pelletier, William R. Walsh, Ken Gall, Dana Carpenter

**Affiliations:** Department of Mechanical Engineering, University of Colorado Denver, 1200 Larimer St, Denver, CO 80204; Prince of Wales Clinical School UNSW Sydney, Surgical and Orthopaedic Research Laboratories (SORL), Kensington 2031, Australia; Pratt School of Engineering, Duke University, Durham, NC 27708; Department of Mechanical Engineering, University of Colorado Denver, Denver, CO 80217-3364

**Keywords:** finite element modeling, osseointegration, gyroid, porous metallic material, bone implant assembly

## Abstract

Creating the optimal environment for effective and long term osseointegration is a heavily researched and sought-after design criteria for orthopedic implants. A validated multimaterial finite element (FE) model was developed to replicate and understand the results of an experimental in vivo push-out osseointegration model. The FE model results closely predicted global force (at 0.5 mm) and stiffness for the 50–90% porous implants with an *r*^2^ of 0.97 and 0.98, respectively. In addition, the FE global force at 0.5 mm showed a correlation to the maximum experimental forces with an *r*^2^ of 0.90. The highest porosity implants (80–90%) showed lower stiffnesses and more equitable load sharing but also failed at lower a global force level than the low porosity implants (50–70%). The lower strength of the high porosity implants caused premature plastic deformation of the implant itself during loading as well as significant deformations in the ingrown and surrounding bone, resulting in lower overall osseointegration strength, consistent with experimental measurements. The lower porosity implants showed a balance of sufficient bony ingrowth to support osseointegration strength coupled with implant mechanical properties to circumvent significant implant plasticity and collapse under the loading conditions. Together, the experimental and finite element modeling results support an optimal porosity in the range of 60–70% for maximizing osseointegration with current structure and loading.

## Graphical abstract

**Figure:**
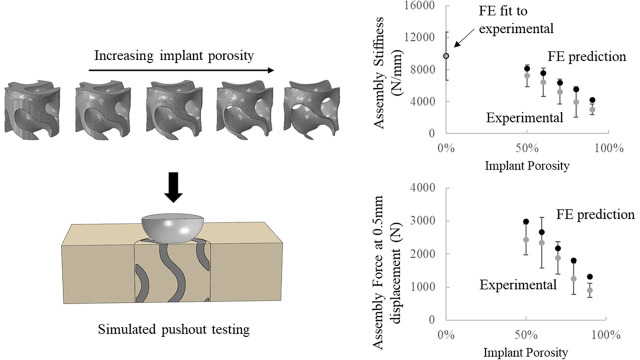

## Introduction

New bone formation at the bone–implant interface is a critical factor for fixation and a long-term successful clinical outcome. This is commonly achieved through osseointegration, the direct connection between bone and implant [1,2]. Osseointegration is a well-proven approach to create a mechanical environment that provides appropriate stress transfer from the implant to surrounding bone to assure early stimulus for bone formation, remodeling, and, finally, maintenance of bone stock [1,3]. Stress shielding occurs when an implant and bone are loaded in parallel, and the implant transmits a majority of the load due to its significantly higher stiffness [4,5]. Due to bone's constant adaptation and remodeling based on loading, it will be resorbed [6–8]. Increased resorption can lead to implant loosening and increase the risk of bone and implant fracture. To minimize or prevent stress shielding, a common approach is to improve load sharing between the implant and bone by reducing implant global stiffness by changing the material, geometry, as well as porosity [9–12]. Understanding the mechanical environment of the bone surrounding an implant and optimizing it to encourage healthy mineralization and maintenance would provide valuable insight to consider in implant design and development.

The introduction of additive manufacturing has allowed for the incorporation of complex geometric lattices resulting in porous implants that can be finely tuned to reduce implant global stiffness using metallic materials [13]. A benefit of using these additively manufactured lattices over other more traditionally manufactured structures is the ability to produce implants that more closely replicate native bone geometry [14,15]. Lattice structures used include cube, diamond, dodecahedron, octet, and triply periodic minimal surface unit cells (TPMS) [16]. The TPMS class of lattices are defined by adding volume around the triply periodic minimal function class and their zero-mean curvature. They are well suited to orthopedic applications due to their superior strength to weight ratio, high fatigue strength, and mass transport properties for osseointegration [17–19].

While there are many components of TPMS structures that can be adjusted to realize different mechanical behaviors of the structure, the predominant variable is the porosity or void fraction of the structure. For the gyroid lattice, which is a specific type of TPMS lattice investigated in the present study, the porosity can be adjusted by adding thickness to the mathematical function which describes the sheet behavior [19]. Understanding the implications of implant porosity on the bone-implant construct is a crucial component of implant design.

Finite element analysis (FEA) is a useful tool for comparing designs and investigating the influence of different design elements without the material and time cost of benchtop testing and manufacturing. In addition, element-level details can be more easily extracted to understand the local stress and strain fields in the materials during deformation leading up to ultimate failure. Here, finite element modeling has been used to reproduce a well-known experimental animal osseointegration model [20–24].

This study seeks to create and validate a model of the bone-implant assembly to simulate the experimental test setup and predict stiffness and force trends for a range of porosities. This model will be used to understand the global and element-level environment of the bone and implant to determine how changing the porosity affects the assembly performance and osseointegration characteristics. Specifically, load sharing between bone and implant, and local stresses/strains will be quantified and compared for each model. The overarching goal of the finite element model is to predict the experimental trends with changing porosity and shed light onto the local deformation mechanisms that are driving the experimental results.

## Methods

**Fig. 1:**
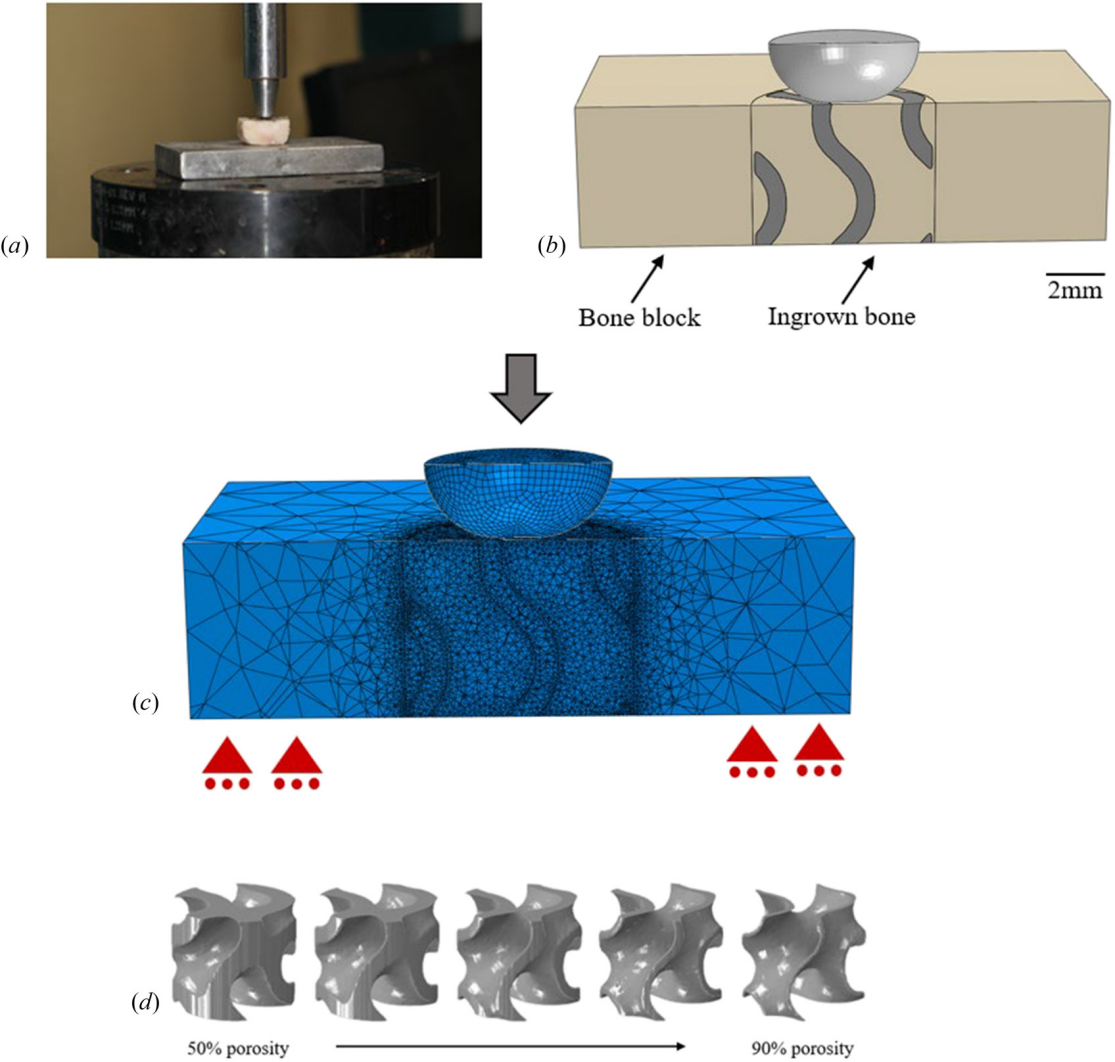
Experimental test setup (*a*). Model setup including outer bone block (beige), inner ingrown bone (beige), implant (dark gray), and indenter (light gray) (*b*). Sample meshed model with illustrated boundary conditions. Roller constraints on bottom surface of the bone block and displacement applied to the rigid indenter (*c*).Implant geometry for each porosity (*d*) scale bar is approximately 2 mm.

Finite element models were constructed of the bone-implant assembly to replicate the published experimental pushout test setup [20]. The experimental data presented are from an ovine bicortical defect model, which tested the strength of osseointegration using a pushout test at the 12 weeks postoperative time point. Each model consisted of an outer bone block, a titanium implant, an ingrown bone region filling the void space of the implant, and an indenter (Fig. 1). Titanium implant porosities investigated in this study were 0%, 50%, 60%, 70%, 80%, and 90%. Each implant had a 6 mm diameter and a height equal to the height of the bone block. The bone block was 18 mm × 18 mm with a 4.95 mm height. The ingrown bone was a cylinder concentric with the implant, comprised of the void space created by the implant. Its height was equal to the height of the bone block and the diameter was 6.2 mm to replicate the region of healing bone tissue surrounding the implant observed in histology imaging [20]. This paper will refer to the inner bone region as ingrown bone, the outer bone region as the bone block, and when bone is being generically referenced it will be referred to as bone (Fig. 1(*b*)). Models used in the development phase were quarter models, meaning the model was cut on both vertical axes through the center of the model and symmetry conditions were employed to reduce computational burden. For this phase of the study, the displacement was applied directly to the top surface of the model.

**Fig. 2:**
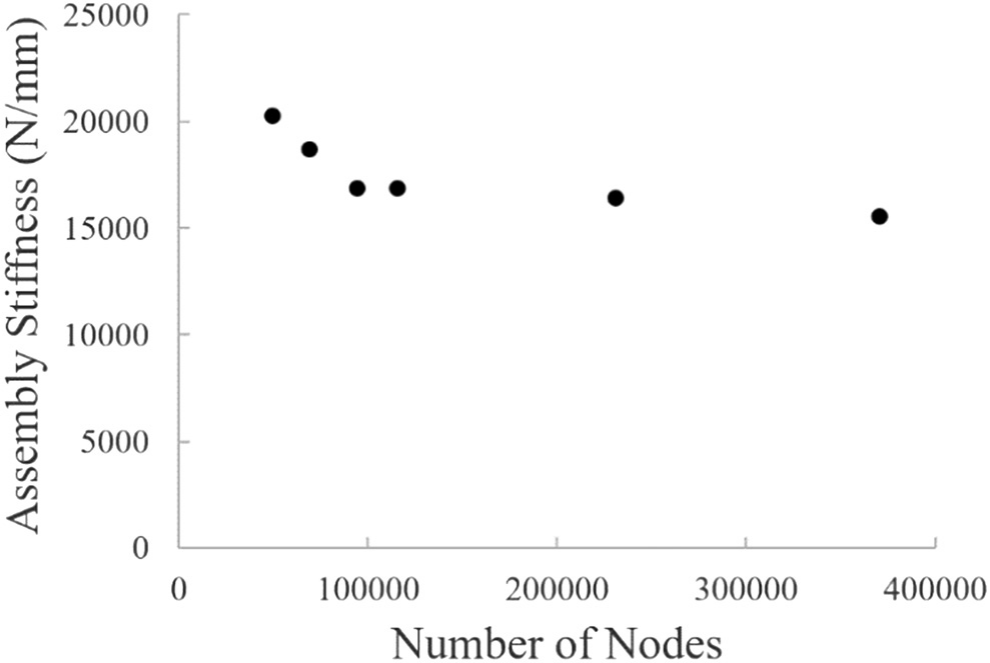
Convergence of assembly stiffness with mesh refinement

Implants with a porous gyroid lattice structure were created in 3matics (Materailise, Leuven, Belgium). The geometry of complete assembly was constructed and meshed in Simpleware (ScanIP). Meshes were constructed from linear tetrahedral elements except for quadrilateral elements used in the indenter. Models had a range of 97,351–170,203 nodes. A sensitivity analysis was performed to ensure the results of the model were independent of mesh size. The model stiffness as a function of total number of nodes for the 70% porous model can be seen in Fig. 2. The mesh was determined to be satisfactory when the stiffness result changed by less than 3% after the number of nodes was increased by 135,000. In addition, the force at 0.5 mm displacement changed by less than 2.5% when comparing the same mesh densities. The sensitivity analysis is assumed to represent the mesh convergence of the remaining porosities. Linear tetrahedral elements were selected because of their ability to capture geometries with high levels of curvature. Elements were more highly concentrated in areas of research interest and geometric complexity, specifically the implant and the ingrown bone. The mesh was coarser in the bone block to strategically prioritize computational efficiency.

### Boundary Conditions.

All experimental data were previously collected and detailed in a previously published study at the 12 weeks timepoint [20]. The data at this time point are assumed to be representative of the early in vivo bone response to implantation. To simulate the experimental pushout test, the bottom surface of the outer bone block was constrained in the vertical direction. Only nodes outside a 4-mm radius from the center of the model on the bottom surface of the bone block were constrained in the direction of displacement to simulate the hole below the assembly in the experiment. A downward displacement was applied to the top, center of the assembly. For the model development phase of the study, the displacement of 0.25 mm was assigned directly onto the top surface of the implant. For the remainder of the study, displacement was enforced on a rigid indenter to more closely simulate the experimental push-out setup. The simulations were run to a calibrated displacement of 0.5 mm to capture the linear response and the onset of plasticity. The contact between the indenter and the rest of the assembly was modeled as frictionless. One node at the corner of the bone block was fixed in all directions to prevent rigid body motion. A tie constraint was used between bone and implant surfaces in contact to prevent relative motion of the surfaces at the interface. The required force was recorded for each displacement increment to output the overall force–displacement curve. Stiffness was calculated by determining the slope of the initial linear region of the force–displacement curve for each sample.

### Material Properties.

**Fig. 3:**
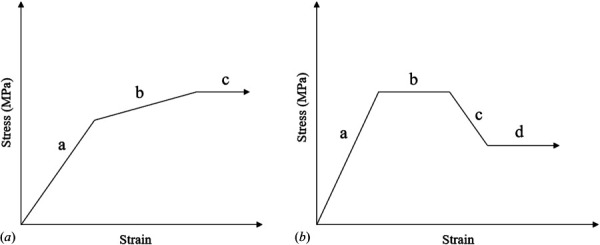
Assumed generic material stress–strain curves for titanium (*a*) and bone (*b*). Titanium stress–strain response behavior from experimental testing, modeled as a piece-wise function. Bone stress–strain behavior developed from previous studies.

To understand the effects of plasticity in individual materials on the assembly behavior, a model development study was implemented. All model components began with linear elastic material properties and capacity for inelastic deformation through plasticity was first added to the implant, then to the outer bone block and finally to the ingrown bone. The material properties of titanium were obtained from experimental testing of the printed solid titanium material and were modeled as a piecewise elastic-plastic function (Fig. 3(*a*)). Experimental testing consisted of tensile testing to failure of dog bone (gauge section diameter: 6 mm, length: 30 mm) samples manufactured in the same manner as the samples investigated in this study. Material property details can be found in Table 1 similar to values used in previous studies [25,26]. This function included an elastic region, defined by the Young's modulus (a), a region of plastic hardening (b), followed by an indefinite perfectly plastic region (c). The material properties of the bone were obtained from the bone modeling development of Keyak et al., which defines a nonlinear stress–strain response based on ash density (Table 2) [27]. Each curve for the bone was composed of four regions. The regions (Fig. 3(*b*)) included the initial linear elastic zone (a), which was defined by the Young's modulus, a plateau at the ultimate stress (b), a softening modulus, (c) and an infinite plateau at the minimum stress postfailure (d). When employing the elastic material properties, each material was defined by the Young's modulus and Poisson's ratio. The outer bone block was assigned a material curve correlating to the upper bound of the ash density spectrum (1.1 g/cm^3^) to reflect that it is fully mature cortical bone. The ingrown bone was assigned an ash density of 0.5 g/cm^3^. This was determined by the calibration outlined in the model calibration section. The Poisson's ratio for titanium was 0.34 and was 0.4 for all bone material properties.

**Table 1:** Titanium material property details

	Titanium
Elastic modulus	125 GPa
Yield stress	896.3 MPa
Ultimate stress	1123.2 MPa
Strain at ultimate stress	0.15

**Table 2:** Bone material property details

	Bone (1.1/cm^3^) ash density	Bone (0.5 g/cm^3^) ash density
Elastic modulus	17.8 GPa	4.1 GPa
Yield stress	121.1 MPa	29.3 MPa
Plateau strain	0.0074	0.0134
Softening modulus	−1000 MPa	−1000 MPa
Minimum stress	51.2 MPa	12.3 MPa

### Model Calibration.

Once the inclusion of plasticity was determined, a rounded rigid body indenter was added to replicate the experimental setup more closely. This indenter had a diameter of 3 mm and a rounded fillet edge of 2 mm radius. The outer bone block was assigned the material property correlating to the fully mineralized cortical bone as noted above. The ingrown bone material property was determined by fitting the maximum force from the solid implant model to that of the average maximum force from the experimental data for the solid group. The ash density selected was 0.5 g/cm^3^. This material property was applied to all ingrown bone in the remaining porosities. This selected value is close to the lowest observed ash density for cortical bone in the material development study, which is a reasonable assumption due to the ingrown bone's incomplete mineralization in this study at the 12-week time point [27,28]. Then a calibration was performed to account for the contributions of experimental artifacts (i.e., test frame compliance, imperfect alignment, and variations in bone geometry) on the global assembly stiffness. A calibration factor (0.000,0924 mm/N) was determined which fit the solid (0% porous) implant model stiffness to the average of the experimental stiffnesses from the solid group. Then, this calibration factor was applied to the remaining porous models in the group. This adjustment added a small displacement to each data point in the finite element results dependent on the force level to replicate the compliance of the experimental test setup and capture all experimental displacement errors using a single calibration value. This resulted in an addition of displacements of between 0.0029 and 0.0392 mm to the finite element results for the increments used to calculate assembly stiffness.

### Data Analysis.

Postprocessing of the finite element models included extracting von Mises stress maps and isolating elements experiencing plastic strain. The average von Mises stress in each component was calculated using the stress value at each element centroid. A traditional load-sharing analysis was performed in which a free body cut was made normal to the displacement direction at the midpoint of the height [6,29,30]. The total force passing through the bone regions (both the ingrown bone and bone block) was summed and compared to the total force passing through the implant. The force summation was done by integrating the stress across the surface of the free body cut to compute the resultant. A linear regression was performed to evaluate the strength of the relationship between the global results, stiffness and force at 0.5 mm displacement, of the FE data and average experimental data.

## Results

**Fig. 4:**
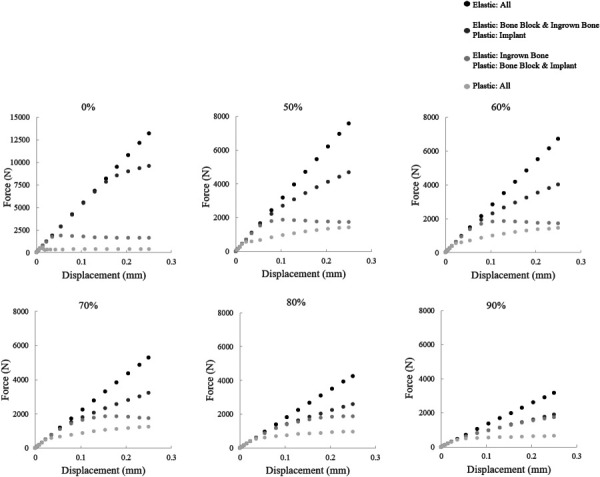
Comparison of assembly force–displacement response as a result of enabling plasticity in each component for all porosities. The included sets are as follows: assemblies with all components modeled with elastic material properties (black), assemblies with elastic material properties used for bone block and ingrown bone and plasticity enabled for the implant (dark gray), assemblies with elastic material properties used in the ingrown bone and plasticity enabled for the implant and bone block (medium gray), assemblies with plasticity enabled for all components (light gray).

The force–displacement curves for each porosity and various combinations of elastic and plastic material constitutive laws are shown below (Fig. 4). The elastic region overlaps for all model variations at small stress and strain values. As the plastic material properties are enabled in various constituents, the deviation from the linear region occurs at a lower force level and the postyield behavior changes. For all models, the fully elastic force–displacement response is drastically different from the fully plastic force–displacement response demonstrating that the contribution of plasticity in each material has a significant impact on assembly deformation and push out behavior. The addition of plasticity in the metallic implant (versus the fully elastic model) results in a slight yield behavior at high force with substantial hardening due to the continued elasticity of the surrounding and ingrown bone. Further addition of plasticity to the surrounding bone block results in a similar yield point in the construct, but a more rapid decline in stress and a softening behavior postyield (relative to the model where only the implant is allowed plasticity). Finally, subsequent addition of plasticity to the ingrown bone (versus elastic only in the ingrown bone) drops the yield point of the construct and results in a moderate hardening as the various materials experience progressive plastic flow at varying rates.

**Fig. 5:**
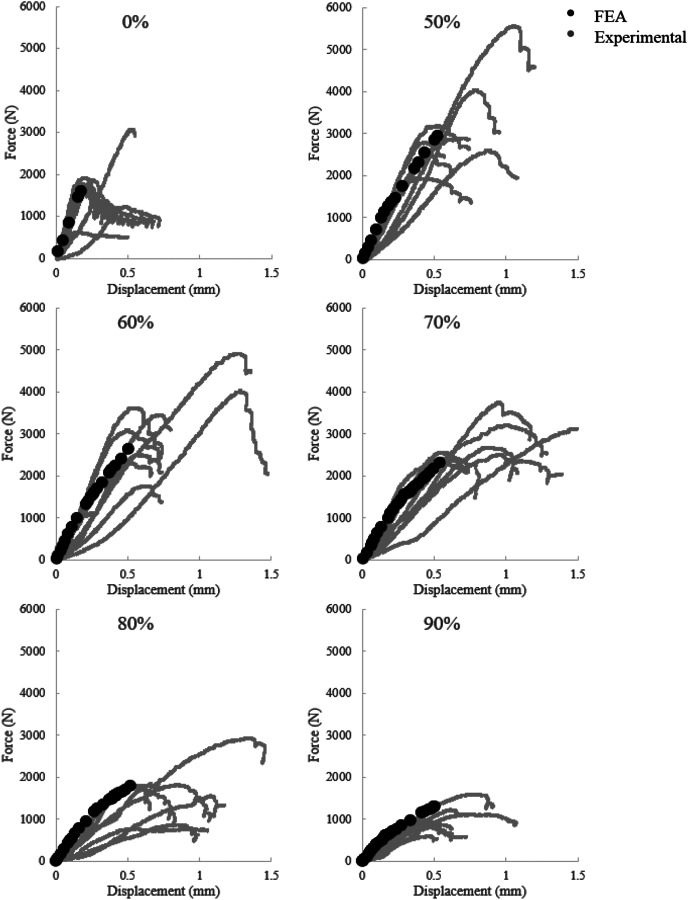
Comparison of the experimental force–displacement results (gray) to the finite element results (black). Finite element data has been calibrated to account for experimental effects, and displacements with 0 N force were also subtracted. Qualitative overlap between FE and experimental results shows initial success of models. Generally, FE curve falls along experimental curves in the elastic region and at the onset of yield.

Full models very closely matching the experimental setup and model geometry were constructed and run using the fully plastic material properties for all components of the assembly. The resulting force–displacement curves for every porosity are shown in Fig. 5. The 0% and 90% porosity models demonstrated the highest and lowest stiffness, respectively.

The finite element model force–displacement data were calibrated to account for systematic experimental errors in displacement measurement and are shown in Fig. 5 compared to experimental data. Displacements with 0 force were also subtracted from overall displacement. Gray curves are the replicate experimental results (*n* = 8), while black curves are the FEA results. The finite element data in the solid (0% porosity) sample represents a fit, while the finite element data in the porous samples (50–90%) are predictions as no experimental force–displacement data from these curves was used in calibrating or defining the model (the geometry of the implant is the only model specific input). Overall, the experimental and simulated results show reasonable qualitative and quantitative agreement. The linear regions in experimental and simulated curves show a slight overestimation from the stiffer finite element models but are overall comparable. For all models, the finite element model correlates well to the elastic region of the experimental data. The finite element model shows a gradual yield as defined by the constitutive relationships similar to the experimental data which shows a mix of elasticity and plasticity followed by sudden fracture.

**Fig. 6:**
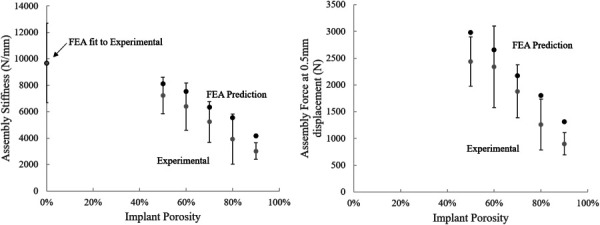
Global parameter comparison between finite element assembly results (solid black) and average experimental (solid gray). Error bars are shown on the experimental results to visualize the standard deviations of the samples. 0% FE model was calibrated to experimental stiffness (hollow black), to predict remaining points in the dataset. FE model predicts trends well, while overpredicting exact values.

Using the calibrated FE curves, global assembly stiffness and the force at 0.5 mm were compared between the FE result and the average values from the experimental data (Fig. 6). FE results for the 0% model (hollow, black data point) were used to fit the simulations to the experimental conditions to predict the results for the remaining porosities (solid, black data points). Average values for experimental results are represented by solid gray data points. The data for assembly force at 0.5 mm for the 0% implant assemblies were not plotted here because this displacement is beyond the displacement at the maximum force. For both assembly stiffness and force at 0.5 mm, the FEA models overpredict the experimental results, as would be expected given the models are idealized and a “best case scenario,” and that not all possible deformation and damage modes are incorporated in the model. The decreasing assembly stiffness with implant porosity is replicated by the FE predicted results.

**Fig. 7:**
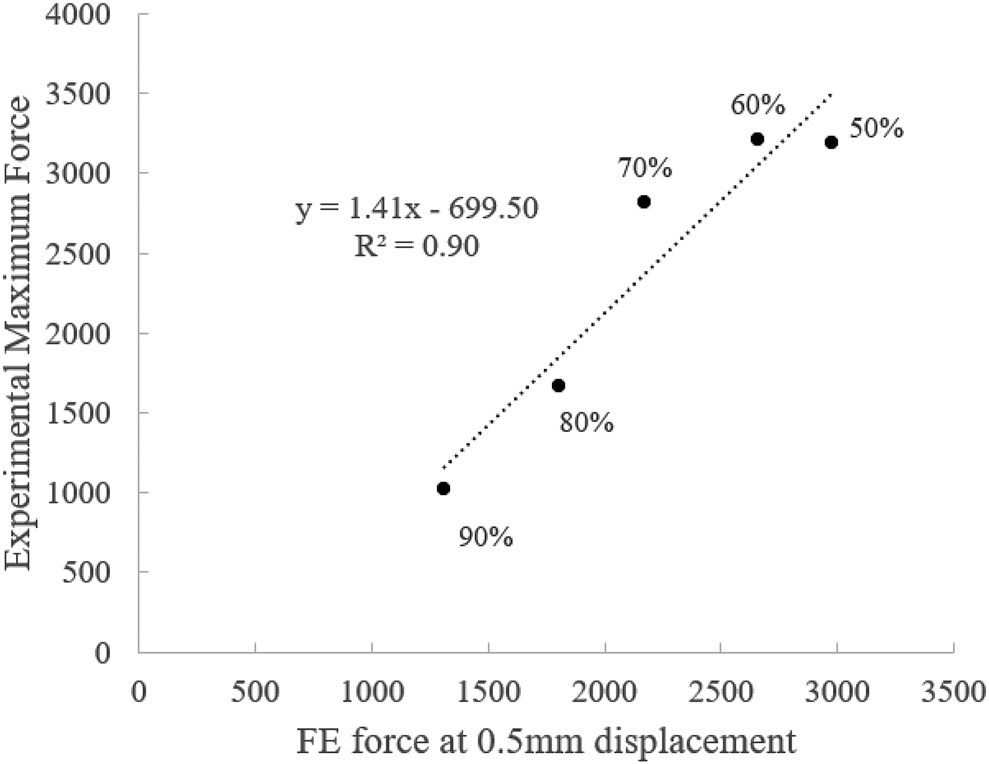
Correlation plot evaluating the ability of the FE force at 0.5 mm to predict the experimental maximum force

The linear correlation between the global parameters of stiffness and force at 0.5 mm displacement was performed to evaluate the strength of the relationship between the predicted FE data (porosities 50–90%) and the experimental results (Table 3). The results of this regression show that the model accurately predicts the experimental trends for assembly stiffness and the force at 0.5 mm for the pushout test. In addition to predicting these trends, the force at 0.5 mm in the FE model is shown to be able to predict the average maximum force of each porosity found in the experimental dataset (Fig. 7).

**Table 3:** Regression analysis results between global parameters from FE simulation and experimental testing

	*r*2	Slope	Intercept	*p*-value
Stiffness	0.98	1.08	−1721.70	0.000,99
Force at 0.5 mm displacement	0.97	1.00	−412.84	0.0025

**Fig. 8:**
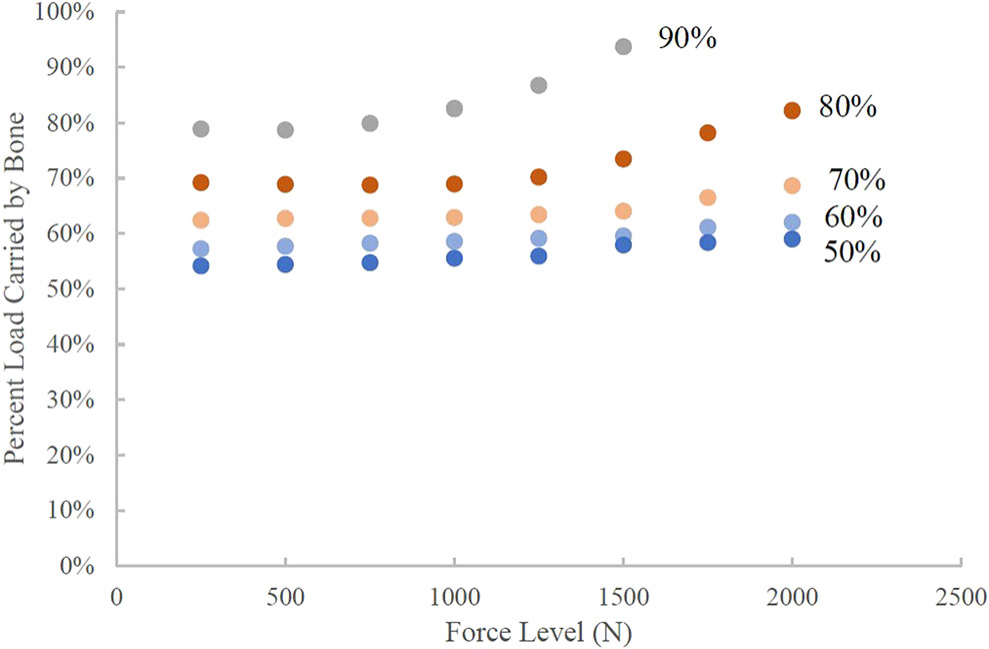
Load sharing results between implant and bone, presented as percent total load carried by the bone at each force level for each porosity. The calculation was performed at the midpoint along the height.

A load sharing (aka stress shielding) analysis was performed to understand the force being transmitted through the bone versus the force being transmitted through the implant at the midpoint of the assembly height. The percentage of the total load transmitted through the bone components (both ingrown bone and mature bone) is plotted in Fig. 8 for all porous samples. The higher the porosity of the implant, the greater force experienced by the surrounding and ingrown bone at all applied force levels. For all assemblies, the percent load carried by the bone increases as the force level increases. The highest porosity assemblies (80% and 90%) show an exponential increase in percent load carried by the bone at the highest force levels. Interestingly, the implants with 50%, 60%, and 70% porosity do not show *substantial* differences in load sharing capacity, all of them result in the bone carrying “approximately” 60% of the applied load within about a ±5% variation. The 80% and 90% porous samples have immediately increased load transfer to the bone at low applied loads followed by substantially higher load transfer to the bone at force levels above 1000 N.

**Fig. 9:**
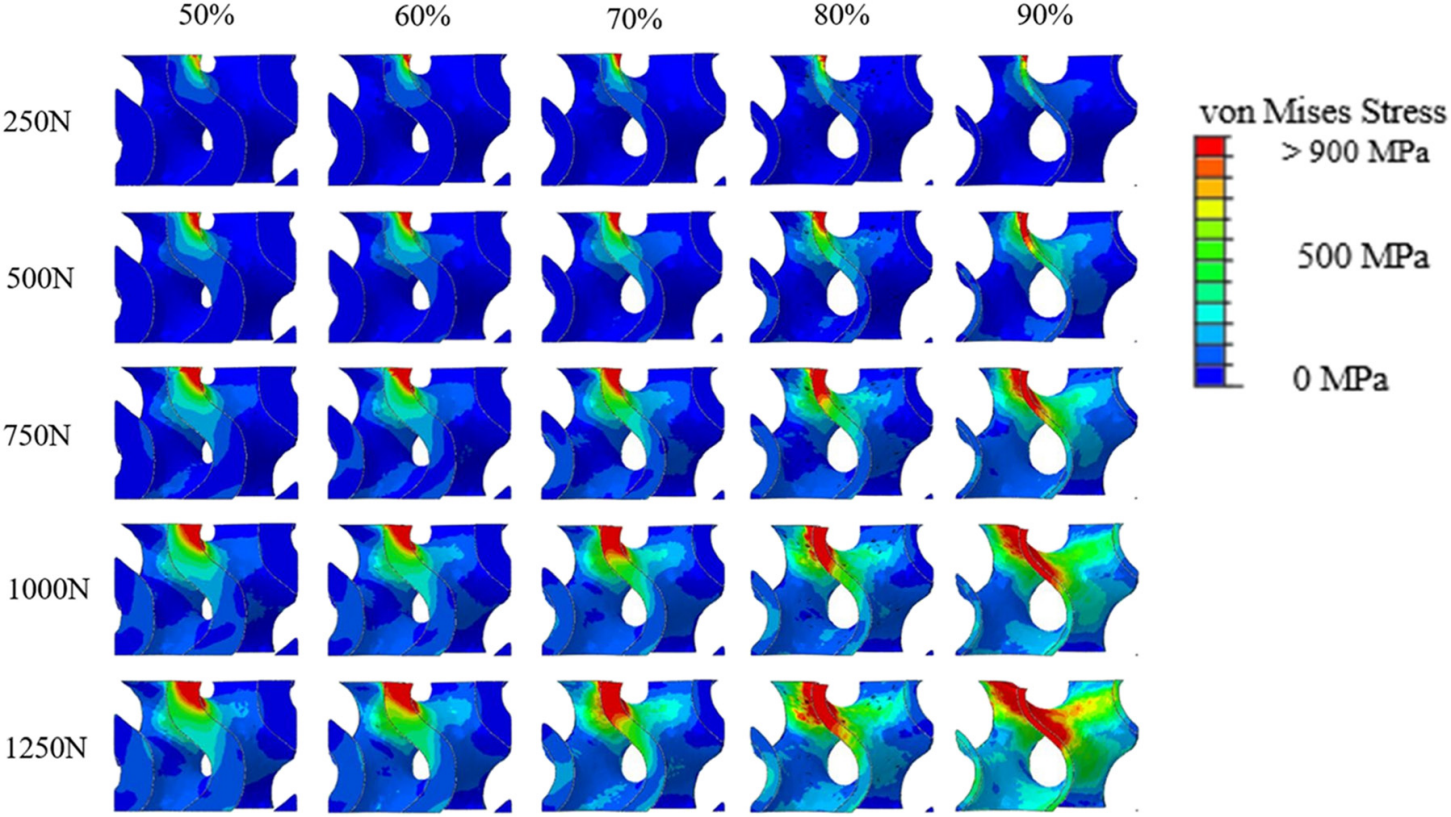
Stress maps for each implant at force levels from 250 N to 1250 N. High-stress regions are initiated at the contact point between the implant and force applicator and propagate throughout structure. 50% implant shows high-stress region to be locally contained whereas 90% implant has a larger spread of high-stress elements.

Stress maps of the isolated implant (bone removed for visualization purposes) with a plane view cut parallel to the applied displacement direction are shown at various force levels in Fig. 9. The legend is formatted so that all elements at a stress level of 900 MPa and above are displayed as red, which correlates to just above the yield stress for titanium resulting in a heat map for implant plasticity. All stress nucleation occurs at the location of contact with the indenter. This view shows the plasticity propagating downward along the vertical component of the gyroid which is closest to the force application location. For higher porosity implants, the stress propagates further along this component down the structure than the lower porosity implants. For all porosities, the span of the plastic zone increases as the applied force level increases. Overall, the stress in the 90% implant is the highest among the group, and the 80% and 90% samples experience the most widespread levels of local yield in the implant during the pushout test.

**Fig. 10:**
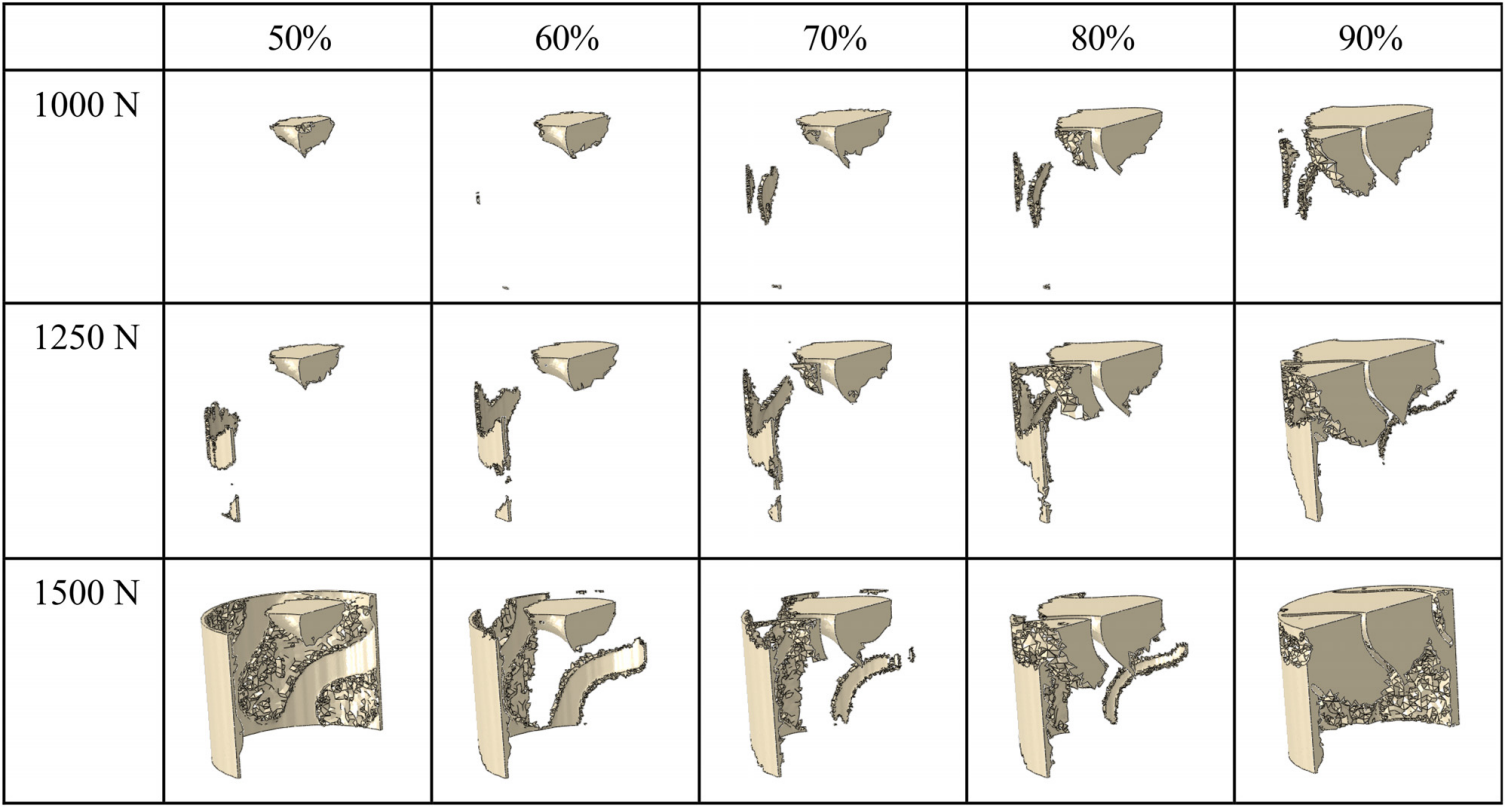
Elements in ingrown bone region experiencing plastic strain at each force level. Plasticity begins in each assembly at contact point with force applicator and emanates from there in addition to plasticity development at interface between ingrown bone outer surface and the mature bone block. The lowest strength model (90%) show the highest amount of plasticity at the interface between the implant and bone, suggesting that this location is the most critical for overall assembly strength.

The deformation and yield of the implant have a significant impact on the yield of both the surrounding and ingrown bone. In Fig. 10, elements in the ingrown bone that are undergoing plastic strain are plotted, while the remaining elements are fully transparent. Force levels of 1000 N, 1250 N, and 1500 N are plotted. Plasticity in the bone initiates in the top center of the ingrown bone where the indenter makes contact with the assembly. For the 1000 N force level, the volume of plastic elements is significantly larger in the 90% porous implant compared to the 50% porous implant. The volume of bone undergoing plastic strain increases as the porosity increases for this force level. At the 1250 N force level, the plasticity spreads downward along the interface between the ingrown bone and mature bone block. The interface is nearly completely plastic for the 1500 N force level for all porosities. The 90% implant assembly shows majority of the void space is experiencing plastic strain at the 1500 N force level. The plastic collapse of the implant along with higher forces in the bone itself causes increased plasticity in the bone that spreads rapidly at lower stresses in high porosity implants.

Examining the split of stresses (versus forces) in the bone and implant reveals data that helps explain the drop in strength with increasing porosity of the implant. Average von Mises stress in the ingrown bone and implant were calculated for each force level from 250 N to 2000 N. The average stress in the ingrown bone is highest for the assembly with the 90% implant until it reaches a peak at 1250 N. From 250 N to 1250 N, the average stress in the ingrown bone increases as porosity increases. The average stress in the implant plots have an exponential shape with the 50% implants having the lowest average stress and the 90% implants having the highest stress at each force level. The data suggest that the local stresses in the implant paint a more complex picture than simply considering the load sharing behavior of the implant and bone. For example, the stresses in the bone and implant are *both* very high in the sample with 90% porosity, and these high local stresses are a driver of early failure. Essentially, the plastic collapse of the implant results in excessive loading on the bone, and despite there being more bone to carry this load the high local stresses result in yield/failure of the implant and bone and ultimately overall construct failure, consistent with experimental data. At lower porosities, the stresses in the ingrown bone and implant both remain lower as the implant maintains its structural strength while still sharing load with the ingrown bone. In these lower porosity implants, overall applied load is effectively more distributed to surrounding bone prolonging collapse of the implant or ingrown bone allowing the stresses to be more distributed throughout.

## Discussion

### Model Development.

The model development phase of this project illustrated the importance of using plastic material properties to fully capture the behavior of the assembly. Often, studies that investigate bone-implant assemblies focus on the elastic regime [6,31]. However, to accurately replicate the trends demonstrated by the experimental dataset, it is imperative to incorporate plastic failure in each material. The postprocessing of modeling results highlights the importance of plasticity in each assembly component, most importantly, the ingrown bone. Plastic strain develops significantly in the ingrown bone prior to the yield point of the assembly (Fig. 10). During the elastic deformation region, the implant and ingrown bone are being loaded as a function of each modulus and the volume it encompasses. However, the ingrown bone is significantly weaker and more compliant than the implant and therefore reaches its failure at an earlier global force level than the implant. This is critical for capturing the experimental behavior, and also clearly demonstrates the consideration of load sharing evolving as a function of global force level. This can be observed in the plastic strain plots of the ingrown bone (Fig. 10). High levels of plastic strain in the ingrown bone are correlated to the likelihood of global failure and are found in the 90% model. There is a large qualitative increase in volume of elements undergoing plastic strain in the 90% compared to the remaining porosities. Without the inclusion of failure in the ingrown bone, the experimental trends cannot be captured.

For the fully plastic assemblies, the global yield force decreases from approximately 2250 N to 700 N for the 50% model from the assembly with elastic bone properties and plastic implant to the assembly with plastic material properties in all components. The global yield force decrease is 600 N–500 N for the 90% model (Fig. 4). This shows that the addition of plasticity to the ingrown bone in the 50% model creates a significantly different assembly performance compared to the plasticity in only the implant. However, the 90% model has a smaller difference in performance when fully plastic compared to plasticity in only the implant. This shows the influence of the implant strength on the assembly. It is a primary determinant and limiting factor of the assembly's overall strength. However, as literature has shown, the bone-implant assembly strength does not dictate the success of an implant alone. The conditions in the surrounding bone and the interface shared with the implant must be conducive to effective load sharing [32–34].

### Comparison to Experimental Data.

The simulated FE models show good overall agreement with the experimental data in both the force–displacement curves collected and the overall performance trends at 12-week time point (Fig. 6). The FE predicted stiffness values followed the expected decreasing trend from 50% to 90% porosity as other studies have reported [4,35,36]. The predicted values in this study remained higher than the average experimental result. A similar outcome is present in the force at 0.5 mm displacement trend. Importantly, many elements at the periphery of the implants with 50% and 90% porosity exhibited loads in the plastic region while those at other porosities did not (Fig. 10). This region dictates the failure load in experimental results and may indicate porosity limits where this interface fails early. Visualizing the location of the plasticity in the bone illustrates the importance of bone at the periphery of the implant, compared to the bone at the center of the implant, supporting the idea that the bone implant interface is a crucial component to bone-implant assembly strength. In addition, this supports the claim that the 60–70% porosity is the optimal design for the gyroid structure due to failure at the interface at porosities above and below this range. The ability to predict the maximum force trends of the experimental data using the FE results at 0.5 mm show that the trends of deformation in the implant and plasticity in the bone are relevant predictors of assembly strength.

The FE prediction follows the same trend as the experimental data but is higher than the experimental result at each data point. There are several reasons for these differences in experimental measurements and model predictions. The FE model uses perfect geometry, which does not represent any imperfections in either the implant or bone, which can lead to crack formation and failure in experimental models. The overall compliance of the testing setup was accounted for, which includes any imperfections in displacement measurements. The same stiffness value of the testing system was applied to each sample and the stiffness trend was maintained which shows this is a systematic effect linearly. Material properties of the ingrown bone for the simulation were calculated from relationships based on cortical and cancellous bone. This was used to represent the 12-week time point which could differ from fully mature bone as it is newly formed and remodeling woven bone. The FE models were assumed to be fully filled with isotropic homogenous ingrown bone with perfect bonding to the surface of the implant. However, this was a simplification made to approximate the experimental models in which the void space was not completely comprised of bone. This could be an additional contributing factor to the overestimation of force. Another simplification made in the FE simulations was modeling the implant and the bone block with equal heights. In the experimental models, the implant protruded upwards above the top surface of the bone block. This could be a source of incongruity between the results, specifically accounting for the plastic strain in the bone directly beneath the applicator in the FE results.

#### Load Sharing.

Load sharing between implant and adjacent bone is often investigated to prevent stress shielding of the bone. This occurs when two materials of differing stiffnesses are loaded in parallel and the stiffer of the two is the predominate bearer of the load [37]. Our analysis quantified the portion of the force passing through each component and provides insight into the load distribution between components through a range of loads including failure. Bone health is dependent on the mechanical environment and stimuli it is experiencing [38,39]. Therefore, the optimization of load sharing as seen in the current model provides valuable information regarding the likelihood of success and are useful from a design point of view. From an overall perspective, the classical load sharing analysis results are expected; the lower porosity implants have higher global stiffness and thus result in the bone carrying less of the applied loads compared to higher porosity implants. However, in terms of understanding the overall behavior of the metal-bone construct, it is insightful to examine the local stress distributions in both the bone and metallic implant beyond the split of forces carried through the implant and bone.

Typical load sharing analyses are performed on assemblies with linear material properties or in the elastic region. This can provide useful information regarding load transmission at low loads. Carpenter et al. compared the load sharing characteristics at different time points (representing different levels of bone ingrowth) for two different materials of the same implant. They show that the less stiff implant results in higher load sharing through the bone [6]. Shum et al. combined FE and animal modeling demonstrated that a tibial tuberosity advancement in a canine with a lower stiffness could promote early bone formation [31]. While only one material was evaluated in our study, the results are in line with these works as at low force levels, the expected trend of an assembly with a higher porosity implant (lower bulk modulus) will have a larger force transmitted through the bone. However, an important distinction is the change in percent force transmitted by the bone as global force increases. For the lowest porosities, specifically 50% and 60%, the percent force transmitted by the bone remains relatively constant as global force increases. On the other hand, for the 80% and 90% assemblies, the percent load transmitted by the bone shows a parabolic shaped curve which increases as global force increases (Fig. 8). This shows that percent load transmission as a function of global force level provides deeper insight into the assembly performance and characteristics. Some studies may argue that matching the stiffness of the implant to the surrounding bone is the best way to reduce stress shielding and therefore promote bone growth [7,36,40,41]. However, when using the elastic region only, this ignores the detrimental effect of the implant's strength on the overall assembly strength. This is shown in the load sharing relationships seen in this study, where the failure of the 90% implant causes a significant increase in percent load transmission through the bone.

It is also notable that the decrease in implant material from the 50% implant to 70% implant increases the percent load transmission through the bone by less than 10%. This indicates that there could be a threshold of implant stiffness/strength that reduces or prevents the excessive loading of the surrounding bone due to high deformation in the implant. Because the load sharing characteristics are similar in the 50%, 60%, and 70% implants, other factors can be used to determine which should be used in device applications. This may include permeability of structure, which influences bone ingrowth to fill the entirety of the implant void [42].

#### Stiffness and Strength Both Contribute to Osseointegration.

**Fig. 11:**
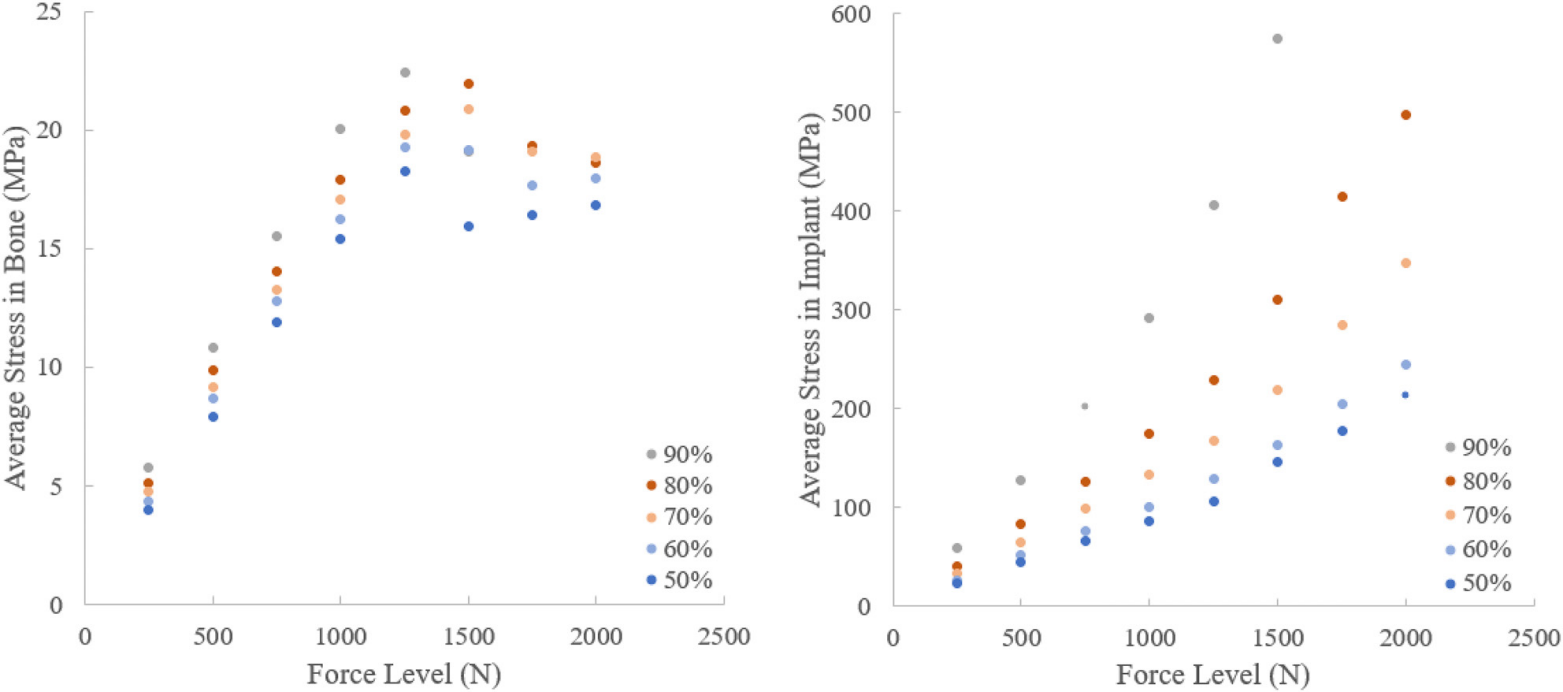
Average von Mises stress in each assembly component, ingrown bone (left), implant (right) at each force level. Assembly with the 90% porous implant has the highest average stress in bone and implant for most force levels. The combination of high stresses in the implant likely results in the high stress in the bone and therefore is a probable cause of the lower assembly strength results previously presented.

The comparison of load sharing at low global force levels reveals the expected trend as a function of porosity, but as the global force increases, a new effect is revealed. This is the effect of implant strength on the mechanical environment of the ingrown bone. The average stress plots show that the 90% implant assembly has the highest average stress in both the implant and the bone (Fig. 11). The high stresses in both components show the connectedness of high porosity implant deformation on high local stresses on the bone. The implant stress maps and elements in the ingrown bone experiencing plastic strain illustrate this effect also. At the 1250 N global force level, the 90% implant shows a significant portion of its volume under high stress. At the same global force level, the same 90% assembly has the largest volume of elements in the ingrown bone experiencing plastic strain (Figs. 9 and 10). This combination effect illustrates that although the higher porosity implants may have the benefit of recreating a similar environment to native bone, by having a lower stiffness, the lower implant strength can negate this benefit in certain loading situations. However, effective bone loading at early timepoints and low force levels is also important to prevent stress shielding [43]. The implant/bone interface must consider the expected level of loading.

The assembly with the lowest porosity has the lowest average stress in both the implant and ingrown bone. The bone in this model does have the lowest amount of elements experiencing plastic strain at 1000 N and 1250 N of global force compared to other porosities (Figs. 10 and 11). This may suggest that the mechanical environment of the bone is not adequate to promote healthy bone growth. However, at these time points, over 50% of the load is being carried by the bone which demonstrates that it is not being totally shielded by the implant. Many studies have investigated the implant as an empty lattice, but this work features the importance of analyzing the implant as a component of the bone-implant assembly [44].

Future studies should investigate the effects of implant porosity on the regeneration and mineralization of bone. This could provide necessary insights into implant design to balance initial fixation and long-term bone health and ingrowth.

## Conclusions

This study successfully shows the finite element model created to simulate the bone implant assembly of porous metallic gyroid implant predicts experimental trends of stiffness and force with porosity from a 12-week preclinical time point. The evolution of load sharing between the implant and bone as force increases demonstrates that the strength and plastic collapse of the implant has a significant effect on increasing the load and stress experienced by the bone. It was determined that to fully model or design an implant's osseointegration environment, both stiffness and strength of the implant should be considered.

## Limitations

As with any finite element model, there are assumptions and simplifications made. These have been addressed in the discussion section. There are also limitations of a large animal model study, including variation in animals and surgical results. Another potential limitation is the data collection at the 12-week time point, which may not reflect long-term ingrowth results. The results of this study apply to the current gyroid structure.

## Data Availability Statement

The datasets generated and supporting the findings of this article are obtainable from the corresponding author upon reasonable request.
